# Identification of protein complexes with quantitative proteomics in S. cerevisiae

**DOI:** 10.3791/1225

**Published:** 2009-03-04

**Authors:** Jesse Tzu-Cheng Chao, Leonard J. Foster, Christopher J. R. Loewen

**Affiliations:** Department of Cellular and Physiological Sciences, University of British Columbia; Department of Biochemistry and Molecular Biology, University of British Columbia

## Abstract

Lipids are the building blocks of cellular membranes that function as barriers and in compartmentalization of cellular processes, and recently, as important intracellular signalling molecules. However, unlike proteins, lipids are small hydrophobic molecules that traffic primarily by poorly described nonvesicular routes, which are hypothesized to occur at membrane contact sites (MCSs). MCSs are regions where the endoplasmic reticulum (ER) makes direct physical contact with a partnering organelle, e.g., plasma membrane (PM). The ER portion of ER-PM MCSs is enriched in lipid-synthesizing enzymes, suggesting that lipid synthesis is directed to these sites and implying that MCSs are important for lipid traffic. Yeast is an ideal model to study ER-PM MCSs because of their abundance, with over 1000 contacts per cell, and their conserved nature in all eukaryotes. Uncovering the proteins that constitute MCSs is critical to understanding how lipids traffic is accomplished in cells, and how they act as signaling molecules. We have found that an ER called Scs2p localize to ER-PM MCSs and is important for their formation. We are focused on uncovering the molecular partners of Scs2p. Identification of protein complexes traditionally relies on first resolving purified protein samples by gel electrophoresis, followed by in-gel digestion of protein bands and analysis of peptides by mass spectrometry. This often limits the study to a small subset of proteins. Also, protein complexes are exposed to denaturing or non-physiological conditions during the procedure. To circumvent these problems, we have implemented a large-scale quantitative proteomics technique to extract unbiased and quantified data. We use stable isotope labeling with amino acids in cell culture (SILAC) to incorporate staple isotope nuclei in proteins in an untagged control strain. Equal volumes of tagged culture and untagged, SILAC-labeled culture are mixed together and lysed by grinding in liquid nitrogen. We then carry out an affinity purification procedure to pull down protein complexes. Finally, we precipitate the protein sample, which is ready for analysis by high-performance liquid chromatography/ tandem mass spectrometry. Most importantly, proteins in the control strain are labeled by the heavy isotope and will produce a mass/ charge shift that can be quantified against the unlabeled proteins in the bait strain. Therefore, contaminants, or unspecific binding can be easily eliminated. By using this approach, we have identified several novel proteins that localize to ER-PM MCSs. Here we present a detailed description of our approach.

**Figure:**
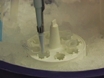


## Protocol

### Materials and Methods

Yeast strains
  All strains used in this study are based on the BY4742 background. The SILAC control strain (*Mat a, his3, leu2, ura3, lys2 and arg4::G418*) was made by mating *arg4* deletion strain (*Mat a, his3, ura3, leu2, and arg4::G418*) to BY4742 (*Mat alpha, his3, leu2, ura3 and lys2*), and the meiotic haploids were obtained by tetrad dissection. Therefore, the control strain is an auxotroph for lysine and arginine. The bait strain was made by PCR-mediated homologous recombination using the vector pBS1479. Therefore, the epitope tag used in this study is the tandem affinity purification (TAP) tag (3).
SILAC:
  The SILAC control strain was grown in minimal dropout media supplemented with 98% L-lysine-4,4,5,5-D4 (0.03 g/L, Cambridge Isotope) and 98% L-arginine-^13^C_6_ (0.036 g/L, Cambridge Isotope).
  The bait strain was grown in regular rich medium with normal isotopic amino acids.
First stage of TAP purification [This protocol is adapted from the Cold Spring Harbor laboratory course manual (4)]**The night before your purification, pre-chill mortar and pestle in -80°C freezer.  Also, pre-chill a beaker in -20°C**  Make these buffers right before use (for 1 L of culture):
  30 ml NP-40 buffer with 25 µl PIC (1/2000), 50 µl of 1M DTT
  20 ml of NP-40 buffer with 1/200 PIC, 50 µl of 1M DTT
  10 ml of TEV-C buffer with 1/2000 PIC, 10 µl of 1 M DTT

#### SILAC and preparation

Grow 1L each of the bait strain and the control strain separately to OD_600_=1.0-1.3.
Pour culture in 500ml Nalgene centrifuge tubes.  Balance the load and pellet cells at 2580X g.
Decant media and resuspend pellet with 50ml of cold dH_2_O while transferring the cells to 50ml centrifuge tubes.  Pellet cells.  **You can freeze the pellet at -80°C**
**important** match the control strain culture to the bait strain culture in 1:1 by dry pellet weight.
Resuspend each cell pellet in 10ml of NP-40 buffer with 1//2,000 Protease Inhibitor Cocktail (PIC).
Mix the two cell cultures by directly decanting one tube to the other. **Grinding**
Pour liquid N_2_ into the pre-chilled mortar and allow it to completely evaporate.  Add 2 ml of cells to the mortar in circular motion.  Pour some liquid N_2_ to freeze the cells.  Grind the cells until the cells become fine powder.  Repeat the process until all the cells are ground.  **Do not allow the cells to thaw. Add liquid N_2_ when necessary**
Transfer the powder to an ice-cold beaker and thaw at room temperature.  When the edges begin to thaw, add 20 ml of NP-40 buffer with 1/200 PIC.
Transfer crude lysate to 40-ml Nalgene tubes.  Spin at 39000 xgfor 30 min.
While waiting, take 300 μl of Sepharose 2B beads (GE).  Wash the beads in 300 μl of NP-40 buffer (1/2,000 PIC) 3 times.  Resuspend the beads in 300 μl buffer so that they are in 1:1 slurry. **Pre-clearing cell lysate**
Transfer the supernatant to a new tube and add the Sepharose 2B beads.  Incubate on a rotating platform in cold room for 30 min.
While waiting, take 300 μl of IgG Sepharose 6 beads (GE). Wash the beads in 300 μl of NP-40 buffer (1/2,000 PIC) 3 times. Resuspend the beads in 300 _l buffer so that they are in 1:1 slurry.
Pellet the beads.  Transfer the supernatant to a new tube.  **Save an aliquot of the cell lysate for Western Blotting later** **Binding to IgG sepharose beads**
Remove the Sepharose 2B beads by centrifuge.  Add the IgG beads (previously prepared in 1:1 slurry).  Separate the contents into two tubes for more efficient binding. Incubate on a rotating platform in cold room for 2 hr.
Spin down the beads.  **Save an aliquot of the unbound faction**
Wash beads with 10 ml NP-40 buffer (1/2,000 PIC).
Wash beads with 3 ml TEV-C buffer (1/2,000 PIC).  **Save an aliquot of the beads.  This will reflect the efficiency of binding** **Eluting from IgG beads by TEV protease cleavage**
Add 5 μl of AcTEV to 1ml of TEV-C buffer (with 1/2,000 PIC).  After mixing, separate the contents into two 1.5 ml Eppendorf tubes for more efficient mixing.  Incubate on a rotating platform in cold room overnight.
Spin down the beads and transfer the eluate to a fresh 1.5-ml tube.  Wash the beads with additional 0.5 ml of TEV-C buffer
Combine the elaute in one tube.  You should have 1.5 ml of eluate in total.  **Save an aliquot of the TEV eluate** **Trichloroacetic acid (TCA) precipatation (For mass spectrometry-based analysis, we recommend using EtOH/ acetate precipitation)**
Adjust aliquots to 25% TCA with 100% TCA.  To do this, separate the 1.5 ml eluate into 2 tubes of 750 μl each.  Add 250 μl of 100% TCA to the tube.  Your solution should turn milky.
Place on ice for 30 min with periodic vortexing.
Spin at maximum speed in the table-top centrifuge in cold room for 30 min.
Wash once with 500 μl of ice-cold acetone containing 0.05 N HCl and spin for 5 min at maximum speed (cold room).
Wash once with 500 μl of ice-cold acetone and spin for 5 min at max (cold room).
Carefully remove acetone and dry pellet.
For silver staining, resuspend the pellet in 50 μl of 1X SDS sample buffer. **EtOH/ acetate precipatation**
Add 20 µg of glycogen
Dilute samples 5X with 100% EtOH and adjust to 50 mM NaCH_3_COO with a 2.5 M stock, pH 5.0
Stand the solution for 2hr
Pellet precipitated protein by centrifugation for 10 min at 12,000 X g at room temperature.

#### NP-40 Buffer (for 200 ml)

**Table:** 

	Stock concentration	Add
10 mM sodium phosphate buffer (pH 7.2)	0.1 M	20 ml
150 mM NaCl	2 M	15 ml
1% NP-40	100%	2 ml
50 mM NaF	1 M	10 ml
0.1 mM Na_3_VO_4_	10 mM	2 ml
Volume to 200 ml

ADD before use:

1/1,000 of 1 M DTT
1/2,000 of PIC  (Protease Inhibitors Cocktail)

#### TEV-C Buffer (for 50 ml)

**Table:** 

	Stock concentration	Add
25 mM Tris (pH 8.0)	100 mM	12.5 ml
150 mM NaCl	2 M	3.75 ml
0.1 % NP-40	100 %	50 _l
0.5 mM EDTA	500 mM	50 __l
Volume to 50 ml

ADD before use:

1/1,000 of 1M DTT
1/2,000 of PIC

## Discussion

The aliquots saved during the purification procedure should include (1) pre-cleared cell lysate, (2) bound fraction, (3) unbound fraction, and (4) eluted fraction. We recommend analyzing the protein contents of the above aliquots by Western blotting using anti-TAP antibody or silver staining to reflect the binding and eluting efficiency of the experiment. Examples of a silver stained gel and a blot are shown in Figure 1.

Since SILAC provides us with unbiased and quantified measurements of protein binding partners, we only do the first stage of the TAP purification to minimize sample loss. If you experience significant amounts of unspecific binding, you may wish to carry out the entire protocol, which can be found in the Cold Spring Harbor manual (4).

## References

[B0] Loewen CJ Inheritance of cortical ER in yeast is required for normal septin organization. Jour. Cell Biol.

[B1] Ong S, Foster LJ, Hoog CL, Mann M Mass spectrometric-based approaches in quantitative proteomics. Methods.

[B2] Puig O The Tandem Affinity Purification (TAP) method: a general procedure of protein complex purification. Methods.

[B3] Amberg DC, Burke DJ, Strathern JN Methods in Yeast Genetics.

